# Uncovering Urinary Proteome Differences in Very Preterm Infants with and without Preterm Brain Injury

**DOI:** 10.1021/acs.jproteome.5c00392

**Published:** 2025-09-09

**Authors:** Magdalena Zasada, Maciej Suski, Marta Olszewska, Aleksandra Kowalik, Natalia Łapińska, Weronika Pogoda, Przemko Kwinta

**Affiliations:** † Department of Pediatrics, 49573 Jagiellonian University Medical College, Wielicka 265 Street, 30-663 Krakow, Poland; ‡ Department of Pharmacology, Jagiellonian University Medical College, Faculty of Medicine, Grzegórzecka 16 Street, 31-531 Krakow, Poland; § Proteomics Laboratory, Centre for the Development of Therapies for Civilization and Age-Related Diseases CDT-CARD, Jagiellonian University Medical College, Skawińska 8 Street, 31-066 Krakow, Poland; ∥ Department of Pharmaceutical Technology and Biopharmaceutics, Jagiellonian University Medical College, Faculty of Pharmacy, Medyczna 9 Street, 30-688 Krakow, Poland

**Keywords:** prematurity, urinary proteome, preterm brain injury, intraventricular hemorrhage, periventricular white matter injury, proteoglycans, fatty acid-binding proteins

## Abstract

Premature infants are at high risk for brain injuries such as intraventricular hemorrhage and periventricular white matter injury. This study applies omics technology to analyze urinary protein expression, aiming to clarify preterm brain injury mechanisms and identify therapeutic targets. Urine samples were collected from 29 very preterm infants (VPI) without brain injury and 11 with moderate/severe injury at eight time points: Days 1, 2, 3, 4, 6, 8, 28, and term-equivalent age (TEA). Brain damage was assessed using the Kidokoro scale and MRI at TEA. SWATH-MS and bioinformatics were used to identify differentially expressed urinary proteins and affected pathways. Fifty-six proteins showed significant expression differences. Notably, extracellular proteoglycans (NCAN, ACAN, BCAN), associated with neuroprotection, were markedly reduced in infants with brain injury. Conversely, fatty acid-binding proteins (FABP1, FABP3, FABP4, FABP7) decreased over time in uninjured infants but increased in those with brain injury, suggesting a role in exacerbating damage. In summary, the urinary proteome of VPI with moderate/severe brain injury differs significantly from those without injury. Reduced neuroprotective proteoglycans and elevated FABPs highlight potential molecular markers and targets for intervention in preterm brain injury.

## Graphical abstract

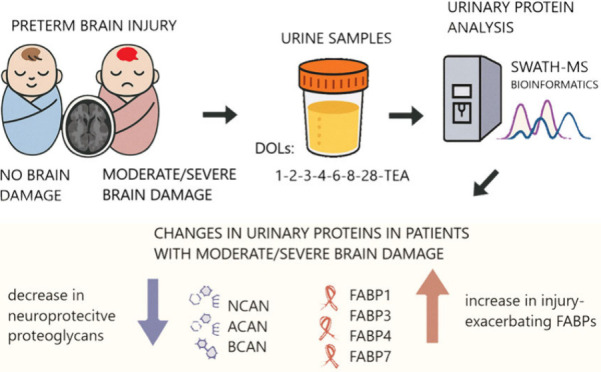

## Introduction

Recent advancements in medicine have significantly improved survival rates for very preterm infants (VPI). However, preterm birth often leads to damage to the developing brain. Periventricular white matter injury (PWMI) and intraventricular hemorrhage (IVH) are the most common forms of brain injury in premature infants. Despite significant advancements in neonatal intensive care, these injuries remain a major cause of both mortality and long-term morbidity, frequently leading to lifelong neurological complications. The underlying mechanisms that drive the onset, progression, and recovery of preterm brain injuries - as well as their impact on the continued development of the immature brain - are still not fully understood.

Urinary proteomics provides a novel, noninvasive approach to investigating cellular processes and functions that may influence susceptibility, progression, diagnosis, and treatment of various disorders. Under normal physiological conditions, about 70% of urinary proteins originate from the kidneys, while the rest come from peptides and small proteins filtered from the bloodstream or secreted into the urine.^1^ As a result, urinary proteomics can serve as an effective reflection of the health status of both the kidneys and other organs, including the central nervous system.^2^ Studies have shown that various neurological conditions, including neurodegenerative^3^ and neuropsychiatric disorders,^4^ stroke,^5^ meningitis,^6^ and multiple sclerosis,^7^ are linked to changes in urinary protein levels. In the context of neonatal neurological disorders, proteomic analysis of animal models of hypoxic-ischemic encephalopathy (HIE) has revealed notable alterations in plasma protein levels.^8^ Recently, Gurtoo et al.^9^ performed a proteomic analysis of the serum and urinary samples collected within 24 h of birth from 38 newborns with HIE. They identified several promising biomarkers - APOD, ORM1, SOD1, and FABP1 - associated with pathways involved in amyloid fiber formation, programmed cell death, reactive oxygen species detoxification, and neurodegenerative diseases. These biomarkers may aid in predicting the severity of neonatal asphyxia. Moreover, both urinary AGT and FABP1 may serve as potential biomarkers for the early diagnosis of HIE.^10^ However, no comprehensive proteomic analysis has been performed in infants with preterm brain injury using either serum or urine. Exploring the urinary proteome in preterm infants with cerebral injury could offer valuable insights into protein level changes that reflect the underlying pathological processes and recovery mechanisms in the brain and other organs.

The aim of this study was to identify regulated urinary proteins and altered functional pathways in VPI with moderate/severe brain damage to improve the understanding of the molecular mechanisms driving preterm brain injury.

## Methods

### Study groups

This prospective, single-center study was carried out in the neonatal intensive care unit (NICU) at the Institute of Pediatrics, Jagiellonian University Medical College, Krakow, Poland, between April 2021 and July 2023. Infants born at <32 weeks of gestational age (very preterm infants, VPI) and admitted to the NICU within the first 24 h of life were enrolled in the study. The exclusion criteria were: (1) congenital heart or kidney abnormalities, or any structural brain anomalies identified on cranial ultrasound at admission; (2) multiple pregnancies; and (3) clinical suspicion of metabolic or genetic disorders. The study received approval from the local ethics committee, and written informed consent was obtained from the infants’ parents. Perinatal and neonatal demographic and clinical data were collected prospectively.

All VPI underwent brain MRI at term-equivalent age (TEA), as previously described.^11^ MRI images were scored by two independent investigators for the presence of brain injury via the Kidokoro scoring system.^12^ Patients were grouped based on their global brain abnormality score (GBAS). For further analysis, infants with a GBAS indicating normality (= no brain injury group) and those with a GBAS indicating moderate to severe brain injury (= moderate/severe brain injury group) were included.

### Urine Collection and Processing

Urine specimens intended for proteomic analysis were obtained at multiple intervals: days of life (DOLs) 1, 2, 3, 4, 6, 8, 28, and at TEA. Noninvasive sampling was achieved by placing sterile cotton balls (Paul Hartmann, Pabianice, Poland) inside disposable diapers; urine was then extracted using sterile syringes (B. Braun Medical AG, Sempach, Switzerland) and transferred into sterile polystyrene tubes (FL Medical, Torreglia, Italy). In cases where urine was collected via sterile urine bags (Zarys, Zabrze, Poland) for clinical indications, aliquots were similarly drawn into sterile vials using syringes. Any samples contaminated with fecal matter were excluded and recollected. Immediately following collection, urine samples were concentrated by centrifugation at 2600 × g for 10 min at 4 °C using Vivaspin Turbo 4 centrifugal concentrators with a 3 kDa molecular weight cutoff (Sartorius, Göttingen, Germany), then stored at – 80 °C until analysis.

### Preparation of samples for LC–MS/MS Analysis; Liquid chromatography–tandem mass spectrometry; Raw MS data Analysis; Spectral library generation and SWATH quantitation; Sample preparation for LC–MS/MS Analysis; LC–MS/MS acquisition; MS data processing and quantification

The methodological details of the procedures described above have been outlined in our previous publication.^13^ In brief, urine proteins were isolated via acetone precipitation and solubilized in SDS/DTT-containing buffer. Protein concentrations were measured using the WF assay.^14^ For digestion, 70 μg of protein underwent the FASP protocol^15^ using Microcon filters (Merck, Darmstadt, Germany), followed by overnight enzymatic digestion with a LysC-trypsin mix (Promega, Madison, WI). Peptides were desalted, concentrated, and pooled for spectral library generation via high-pH reversed-phase fractionation. Prior to LC–MS/MS, all peptides were diluted in 0.1% formic acid and spiked with iRT peptides for retention time normalization.

For spectral library generation, fractionated peptides were separated using nanoLC and analyzed on a TripleTOF 6600+ system spectrometer (Sciex, Framingham, MA) in DDA mode. SWATH-MS was performed on individual samples using a microflow LC setup and variable window acquisition. Detailed gradients and instrument parameters were applied for both modes.

DDA data were searched against the UniProt human database using Spectronaut’s Pulsar engine^16^ with a 1% FDR for peptides, proteins, and PSMs. Deep learning-assisted iRT regression was applied with an R^2^ threshold of 0.8. The library was built using 3–6 fragments per precursor. SWATH data were analyzed using the same software with 1% FDR filtering. Protein grouping was performed using the ID picker algorithm.^17^ Differential abundance was tested using *t* tests with Storey’s multiple testing correction.^18^ The mass spectrometry proteomics data have been deposited to the ProteomeXchange Consortium via the PRIDE^19^ partner repository with the data set identifier PXD064065. Functional enrichment analysis was conducted via ClueGO^20^ in Cytoscape^21^ using PINE^22^ and curated pathway databases. Enrichment was tested using Bonferroni-corrected geometric statistics.

### Statistical Analysis

Categorical variables were summarized as numbers and percentages. Continuous variables were tested for normality (Shapiro–Wilk) and presented as mean ± SD or median (Q1–Q3) as appropriate. Fisher’s exact test, Student’s *t* test, or Wilcoxon test were applied. A p-value <0.05 was considered statistically significant. Analyses were conducted using JMP version 17.1.0.

### Results

A total of 40 VPI were included. Twenty-nine infants with a GBAS indicating normality formed the no brain injury group, and 11 infants with a GBAS indicating moderate to severe brain injury formed the moderate/severe brain injury group. The demographic and hospitalization data of the patients are presented in Table 1.

**Table 1:** Cohort Demographics of All Patients in the Urine Proteomic Study^a^

	No brain injury (*n* = 29)	Moderate/severe brain injury (*n* = 11)	P value and test used
Gestational age (weeks, mean (±SD))	29.0 (±1.9)	28.0 (±1.5)	0.1153 ^T^
Sex (# F/# total (% of F))	15/29 (51.7%)	4/11 (36.4%)	0.4882 ^F^
Body weight at birth (g, mean (±SD))	1336 (±381)	1242 (±355)	0.4712 ^T^
Head circumference at birth (cm, median [Q1; Q3]))	27 [26.25; 28.25]	25 [23; 27]	0.1039 ^W^
Body length at birth (cm, mean (±SD))	39.5 (±4.9)	39.2 (±5.8)	0.8675 ^T^
Cesarean section (n, (%))	24 (82%)	6 (54.5%)	0.1028 ^F^
Apgar score (5^th^ min, median [Q1; Q3]))	7 [6; 8]	6 [4; 7]	**0.0363** ^**W**^
Respiratory distress syndrome requiring surfactant therapy (n, (%))	20 (69%)	11 (100%)	**0.0433** ^**F**^
IVH ≥ 3^rd^ grade (n, (%))	0 (0%)	9 (81.8%)	**0.0001** ^**F**^
Early onset sepsis (n, (%))	0 (0%)	3 (27.3%)	**0.0167** ^**F**^
Treated hemodynamic patent ductus arteriosus (n, (%))	5 (17.2%)	4 (36.4%)	0.2268 ^F^
Mechanical ventilation (days, median [Q1; Q3]))	0 [0; 5]	18 [4; 39]	**0.0001** ^**W**^
CPAP/NIV (days, median [Q1; Q3]))	0 [0; 3]	14 [4; 24]	**0.0003** ^**W**^
Low flow oxygen therapy via nasal cannula (days, median [Q1; Q3]))	6 [0; 22.5]	7 [4; 35]	0.2899 ^W^
Late-onset sepsis (n, (%))	2 (6.9%)	6 (54.6%)	**0.0026** ^**F**^
Meningitis (n, (%))	0	0	-
Bronchopulmonary dysplasia moderate or severe (n, (%))	2 (6.9%)	5 (45.5%)	**0.0108** ^F^
Retinopathy of prematurity (n, (%))	6 (20.7%)	6 (54.6%)	0.0563 ^F^
Periventricular leukomalacia (n, (%))	1 (3.5%)	5 (45.5%)	**0.0036** ^F^
Acute and/or chronic kidney injury (n, (%))	0	0	-
Duration of parenteral nutrition (days)	10 [6.5; 13.5]	21 [9; 39]	**0.0045** ^**W**^
Weight z-score at discharge (mean (±SD))	–0.04 (±0.95)	0.32 (±0.69)	0.2000 ^T^
Head circumference z-score at discharge (mean (±SD))	–0.14 (±0.97)	–0.33 (±1.48)	0.6962 ^T^
Length z-score at discharge (mean (±SD))	0.31 (±1.30)	0.10 (±1.73)	0.7180 ^T^

a ^F^ – Fisher’s exact test; ^T^ – Student’s *t* test; ^W^ – Wilcoxon test. P-values in bold indicate statistically significant differences.

The study design scheme and the proteomic analysis workflow are shown in Figures 1A and B, respectively.

**Figure 1:**
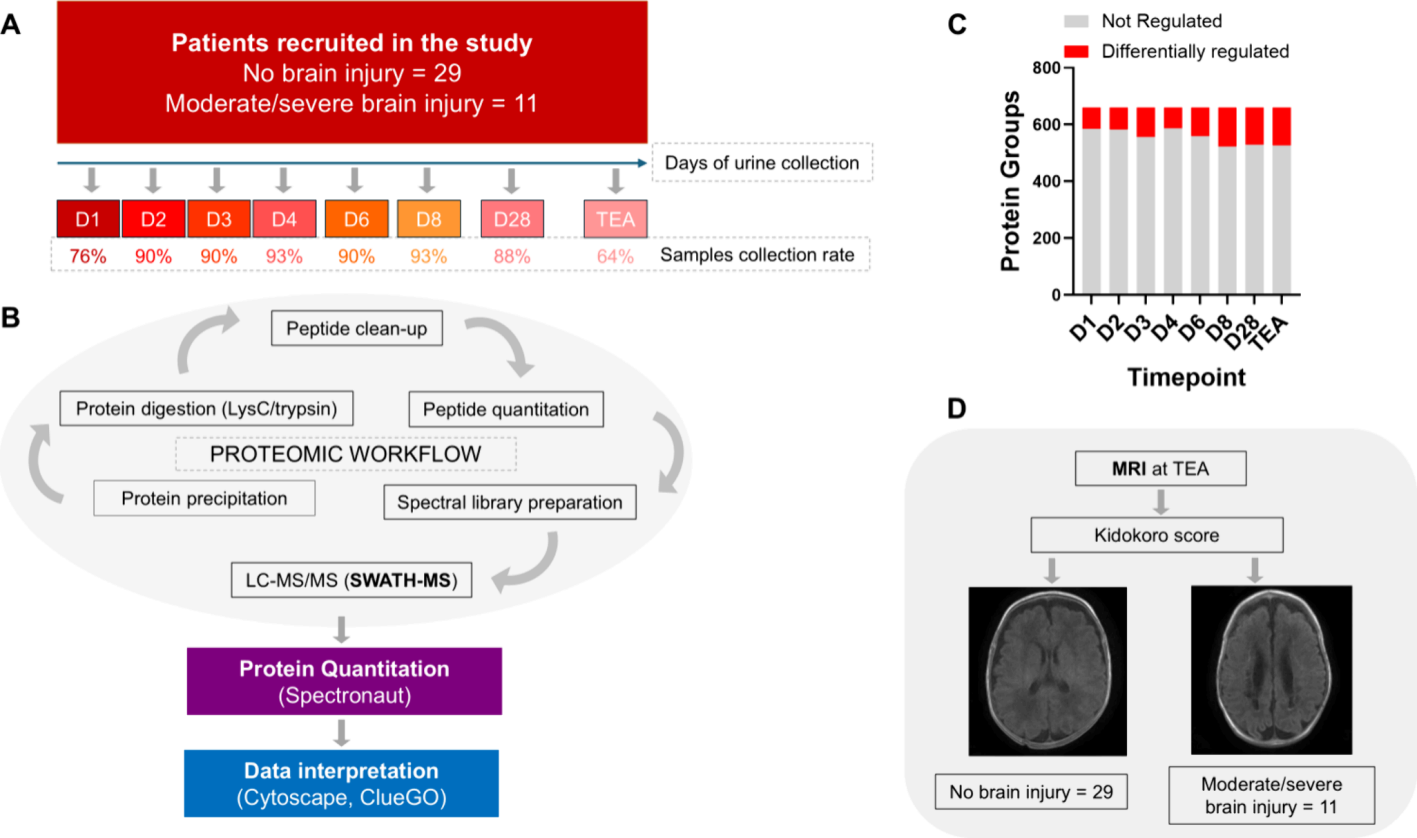
**Overview of the study design and proteomic measurements in neonatal urine**. Schematic description of the study design (A) and the proteomic workflow (B) incorporated to unravel the differences in the urinary proteome of premature neonates. The number of differentially regulated proteins increased during the observation period, ranging from 75 on Day 1 (D1) to 134 at term-equivalent age (TEA) (C). Patients were categorized according to the Kidokoro score. A comparison was made between those without preterm brain injury and those with moderate/severe preterm brain injury (D).

Only proteins that were identified in at least 80% of the collected samples by at least two unique peptides were selected for further quantitative proteomic analysis to create a robust and reliable quantitative data set. For the above-mentioned set of proteins, we performed missing value imputation via a global imputation strategy where the missing values were imputed based on random sampling from a distribution of low-abundance signals taken throughout the entire experiment. This resulted in the generation of a data set of 725 proteins quantified in each sample (Figure 1C). The estimated significant absolute fold change cutoff was set at 2.0 to secure the power of statistical testing above the 0.8 threshold. On average, 104 protein groups (ranging from 73 to 138) were differentially regulated on each collection day, spanning from the first day of life to the term-equivalent age (Figure 1C and 2A), and tended to increase during the observation period (Figure 1C, Supplementary Tables S1–S8).

**Figure 2:**
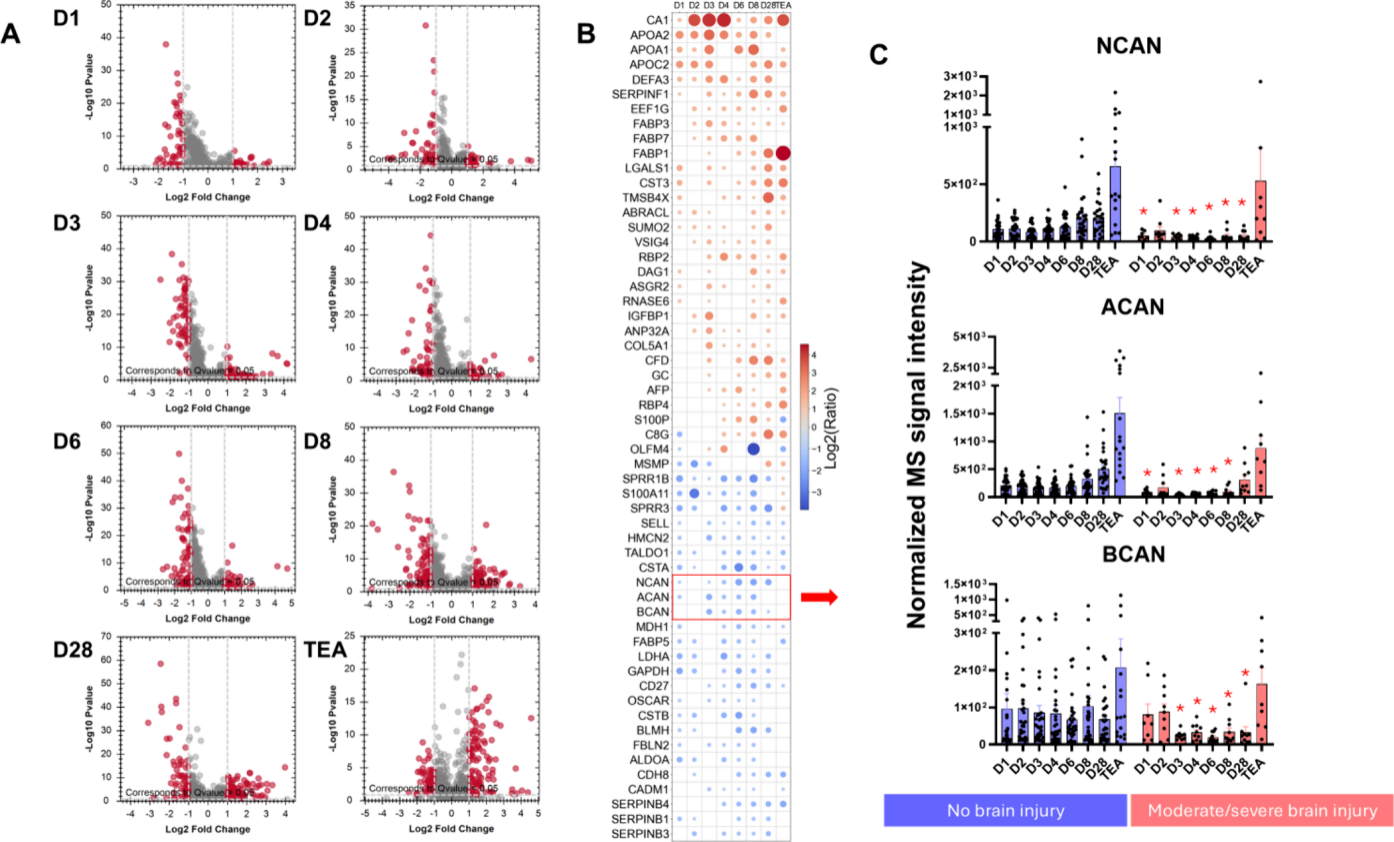
**Regulated urinary proteins and their quantitative time-dependent changes.** The urinary proteome underwent substantial changes over time, with significant differences between infants with moderate/severe brain injury and their counterparts without brain injury at all time points examined (A). A total of 56 proteins in the urine differed significantly between the groups at least 5 out of 8 time points, showing a consistent pattern of being either increased or decreased, while only a few showed different patterns of change over time (B). A family of proteoglycans (including NCAN, ACAN, and BCAN) was significantly repressed in urine samples from the group with moderate/severe brain injury compared with those from the reference group without brain injury (C). The data are presented as bars (means ± SEMs) with individual values; *n* = 24–29 in the reference group without brain injury (blue), and *n* = 8–10 in the group with moderate/severe brain injury (red). Statistically significant differences are indicated (*q* < 0.05).

Interestingly, the urinary concentrations of most of the proteins in the moderate/severe brain injury group were consistently different from those in the corresponding reference group (no brain injury group) during the time course of the study period (Figure 2B). For example, a group of extracellular proteoglycans that bind hyaluronan acid, neurocan core protein (NCAN), aggrecan core protein (ACAN), and brevican core protein (BCAN), were consistently repressed in the moderate/severe brain injury group (Figure 2C). Only several proteins exhibited time-dependent growth patterns and differed during the observation period. For example, fatty acid-binding protein 1 (FABP1), retinol-binding protein 4 (RPB4) and vitamin D-binding protein (GC) were all increasingly upregulated over time in the moderate/severe brain injury group, whereas the urinary concentrations of serpin B4 (SERPINB4) and cadherin-8 (CDH8) followed the reverse trend (Figure 2B). In this context, we demonstrated complex time-dependent changes in carbonic anhydrase 1 (CA1) in the moderate/severe brain injury group. Its urinary concentration markedly increased during the first 4 days of life (mean fold change: 16.95), after which it decreased to a fold change value of 2.62 on Day 6. From Day 8, the urinary level of CA1 increased again until the end of the observation period, from 4.08- to 12.99-fold change relative to the reference group (Figure 2B).

Next, we performed pathway analysis in which the differential abundance of a particular protein was averaged across the time points (considering that the majority of proteins were either consistently repressed or induced during the study time frame). Additionally, for more informative results, we restricted the analysis to only those regulated proteins, which were altered in at least 3 of 8 time points. In this way, we excluded urinary proteins with sparse differential abundance from the analysis. The key functional domains enriched were those related to peroxisome proliferator activated receptor (PPAR) signaling, degradation of the extracellular matrix, IL-18 signaling, hemostasis, and complement and coagulation cascades and were all intensified, as inferred from quantitative changes in the urinary proteome (Figure 3A). Interestingly, as evidenced by the differences in the percentage of associated genes found in a particular pathway, PPAR signaling appeared to decline over time, whereas extracellular matrix degradation and complement cascades intensified during infants observation (Figure 3B).

**Figure 3:**
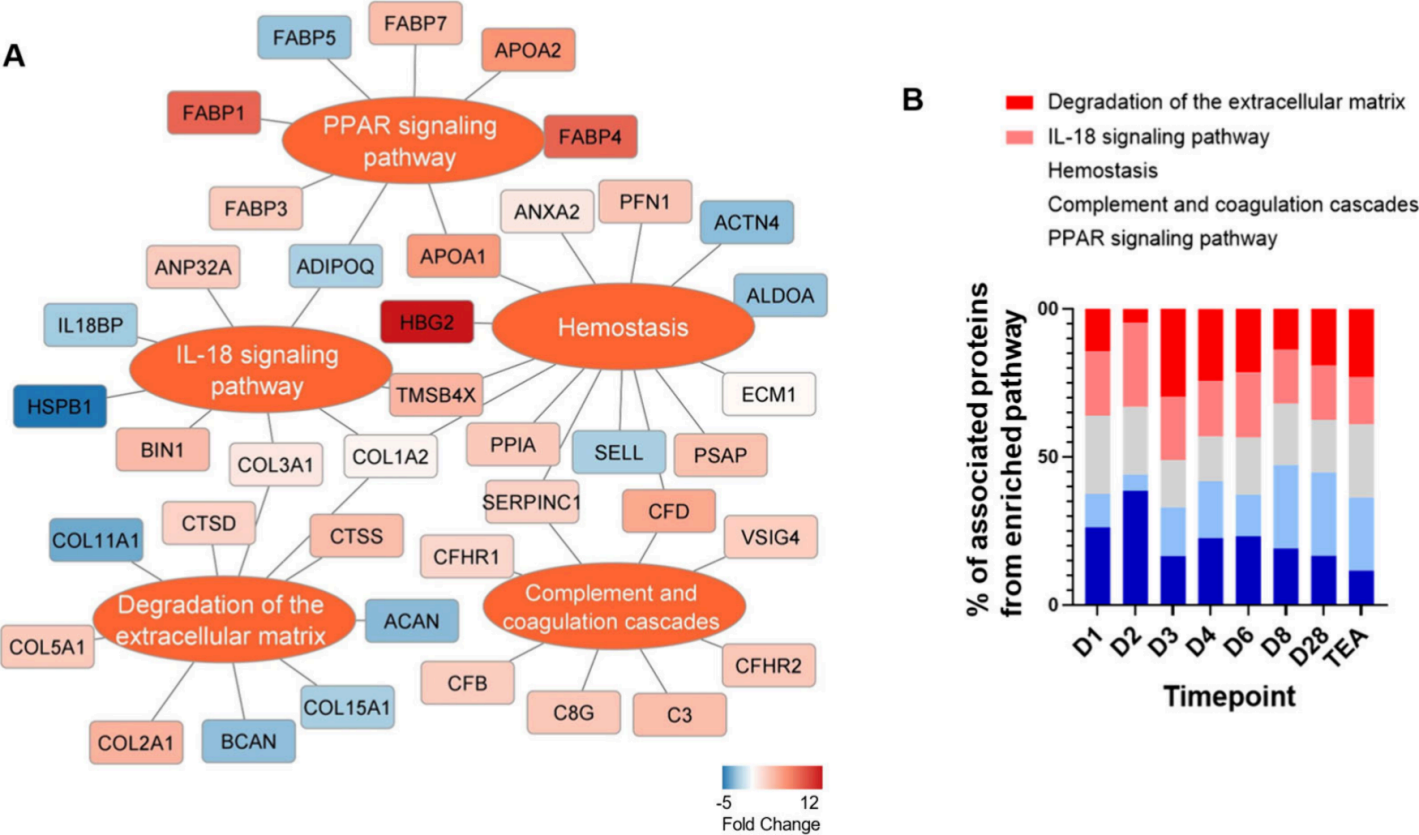
**Functional pathway enrichment of regulated urinary proteins.** Functional pathways revealed by changes in urinary proteins (A). The indicated protein quantitative fold change values were calculated as the means for all time points for a particular protein. For the analysis, the data set was reduced to only those proteins that were significantly differentially expressed at a minimum of 3 of the 8 time points. The percentage of associated genes from the PPAR signaling pathway decreased over time, whereas extracellular matrix degradation and the complement cascade increased during the infant observation period, which suggests functional variations during the observation period (B).

Finally, considering the observed protein quantitative patterns and functional enrichment, we extracted the quantitative traits of a group of fatty acid-binding proteins and reported that the levels of FABP1, FABP3, FABP4 and FABP7 followed a similar pattern of time-dependent differences and decreased over time in the no brain injury reference group, whereas the counterparts of the moderate/severe brain injury group presented a disturbed pattern of changes, resulting in a relative increase in the abundance of FABPs at the analyzed time points (Figure 4).

**Figure 4:**
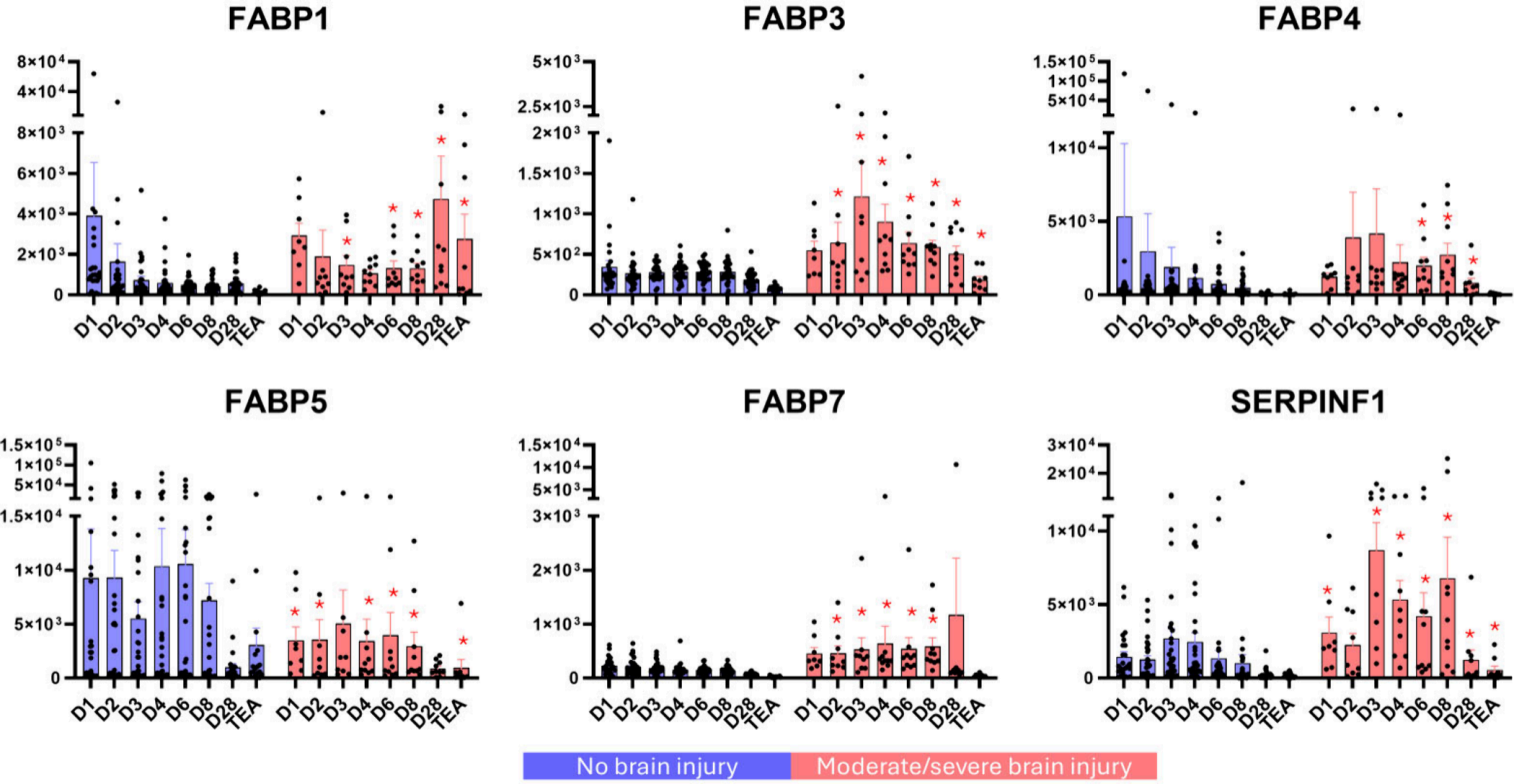
**Quantitative characteristics of selected urinary proteins.** Quantitation of fatty acid-binding proteins (FABPs) and pigment epithelium-derived factor (SERPINF1) at the analyzed time points. The data are represented as bars (means ± SEMs) with individual values; *n* = 24–29 in the reference no brain injury group (blue), and *n* = 8–10 in the moderate/severe brain injury group (red). Statistically significant differences are indicated (*q* < 0.05).

Intriguingly, the FABP5 isoform was the only isoform that followed an opposite trend, where the reference no brain injury group presented a sustained increase in the FABP5 urinary concentration (Figure 4). Conversely, a sustained increase in the SERPINF1 urinary concentration was observed in infants with moderate/severe brain injury (Figure 4).

## Discussion

Our study showed consistent differences in the urinary proteome between infants born before 32 weeks’ gestation who developed moderate/severe preterm brain injury and those who did not. We collected samples at eight time points from birth to TEA. This study is, to the best of our knowledge, the first to examine longitudinal urinary proteomics in relation to preterm brain injury. Brain injury severity was assessed using the Kidokoro scale, which has established prognostic value.^23^

In our study, fifty-six urinary proteins showed significant differences between the groups at least five out of eight time points, indicating a consistent pattern of being either increased or decreased, with only a few showing unique changes over time. Several of the identified proteins are known to be active in the central nervous system.

Patients with moderate/severe brain injury consistently exhibited repression of a group of extracellular proteoglycans that bind hyaluronic acid - namely, neurocan (NCAN), aggrecan (ACAN) and brevican (BCAN). These proteoglycans constitute essential elements of the brain’s extracellular matrix (ECM), forming large aggregates that sequester various proteins, including chemokines, growth factors, and axon guidance molecules.^24^ Notably, they are involved in modulating neuronal adhesion and neurite growth during development, as well as in the differentiation and maturation of the adult nervous system during postnatal development.^25^ NCAN also contributes to the formation of perineuronal nets, which are important for maintaining brain function.^26^ Additionally, Herris et al. reported that serum levels of NCAN and BCAN are linked to brain volume and may contribute to neuroprotection by supporting overall fluid cognitive ability in early adulthood.^27^ With regard to anthropometric measurements at discharge, no significant differences in head circumference z-scores were observed among our study groups. It is plausible that the interval between the initial insult and the time of measurement was insufficient to detect measurable impairment in head growth. However, long-term follow-up and monitoring of head circumference growth are essential. Notably, previous research showed that IVH can lead to proteoglycan buildup in brain tissue.^28^ Our findings may be associated with cerebral damage in the moderate/severe brain injury group and the sequestration of these proteoglycans within the central nervous system. However, the possibility that premature infants with lower concentrations of these proteins may be more vulnerable to preterm brain injury is important to consider.

We also found that cadherin-8 (CDH8) levels gradually declined over time in infants with brain injury. CDH8 is primarily expressed in the brain and is involved in forming key neural circuits.^29^ Alterations in this protein have been associated with autism spectrum disorders,^30^ which is relevant since preterm infants are at higher risk for autism.^31^

Additionally, some downregulated proteins from our pathway analysis seem to reflect processes that occur differently in the brains of VPI with premature brain injury. Heat shock protein family B member 1 (HSPB1) is a chaperone that is essential for cellular defense responses against various stressors, including oxidative stress. In animal models of neonatal hypoxia-ischemia, the upregulation of HSPB1 has been shown to significantly mitigate brain injury and enhance neurological outcomes.^32^ Adiponectin (ADIPOQ), a protein involved in the PPAR and Il-18 signaling pathways that was repressed during the study time frame is a fat-derived plasma hormone with powerful antiapoptotic and anti-inflammatory properties in brain injury, including ischemic and hemorrhagic stroke.^33^ Importantly, ADIPOQ attenuates inflammatory brain injury induced by germinal matrix hemorrhage.^34^

Fatty acid-binding proteins (FABPs) are intracellular proteins playing crucial roles in lipid metabolism, cellular signaling, and energy homeostasis. Among the FABP family proteins, three types (FABP3, FABP5 and FABP7) are specifically localized in the brain, whereas FABP1 is predominantly expressed in the liver. Recently, Gurtoo et al.^9^ reported that serum and urinary FABP1 levels may serve as promising biomarkers of HIE within 24 h after birth. FABPs have recently been linked to important regulators of ischemic stroke.^35^ They mediate a plethora of different regulatory functions (i.e., the upregulation of apoptotic signals, the degradation of tight junctions of blood–brain barrier proteins, the induction of mitochondrial damage and the enhancement of neuroinflammation), which are collectively recognized as aggravating ischemic brain injury.^35^ In this context, the different quantitative pattern of FABP5 remains surprising since it shows functionality like that of FABP7 in exacerbating neuroinflammation.^36^ Whether FABP5 repression exemplifies a regulatory mechanism evoked in the moderate/severe brain injury group to counteract the detrimental effects of FABP changes that drive preterm brain injury remains an attractive hypothesis to be tested.

Moreover, our proteomic analysis provides additional evidence for such mechanisms, for example, an increase in pigment epithelium-derived factor (SERPINF1) in infants with moderate/severe brain injury. This protein helps reduce inflammation, support neurons, and maintain blood–brain barrier integrity, with its dysfunction thought to be a critical contributor to the pathophysiology of various neurological conditions, including ischemic stroke.^37^

Another notable protein was carbonic anhydrase 1 (CA1), a common enzyme in red blood cells. When hemolysis occurs, CA1 is released into the bloodstream, and because of its small size, it crosses the glomerular barrier and appears in the urine. Its fluctuating levels in our study may reflect increased hemolysis in more severely ill infants or the effects of blood transfusions commonly needed in cases of severe IVH.^38,39^

## Strengths and Limitations

The methodology employed in this study is a notable strength. A large number of patient samples were collected during hospitalization, spanning from premature birth to TEA. Additionally, the study has a clearly defined end point. A further advantage is the use of modern, rigorous proteomic analysis in our investigation. However, there are several limitations to consider. The primary limitation of our study was the small sample size, especially in the moderate/severe brain injury group (n = 11), which limits statistical power and generalizability.

Moreover, urine collection at TEA was limited, largely due to early discharge or transfer of some infants to lower-level care facilities. This was expected, as the study was designed to avoid disrupting the standard course of hospitalization. Additionally, some of the urinary proteins analyzed may be present at elevated levels in cases of kidney injury. For example, elevated retinol-binding protein 4 in the urine (RBP4) is a biomarker for proximal tubular dysfunction.^40^ Excessive urinary loss of VDBP has been observed in patients with nephrotic syndrome and type 1 diabetic patients with proteinuria.^41^ Serine protease inhibitor B4 (SERPINB4) may be involved in renal pathologies and could serve as a potential biomarker for kidney dysfunction.^42,43^ Notably, urinary FABP1 may also be a biomarker for impaired proximal tubular protein reabsorption.^44^ While we believe that the proteins in our study are not derived from renal damage, this cannot be entirely excluded. Importantly, no clinical cases of acute or chronic kidney injury were observed among the participants. Furthermore, proteins strongly associated with neuroprotection were predominantly downregulated in the urine of patients with more severe brain injury. This observation supports the hypothesis that individuals with moderate to severe brain injury likely exhibit lower levels of these proteins in the plasma or within the central nervous system, potentially contributing to the pathogenesis and progression of preterm brain injury. Additional evidence, such as serum proteomics, may serve as a valuable tool to validate our findings. Lastly, clinical factors, such as prolonged mechanical ventilation, extended duration of parenteral nutrition, or blood transfusions, may serve as potential confounders, contributing to the proteomic differences observed at later time points. Our results provide preliminary insights, they should be interpreted with caution and confirmed in future studies with larger cohorts.

## Conclusions

Analysis of the urinary proteome revealed that VPI with moderate to severe brain injury showed sustained downregulation of CNS-specific extracellular matrix proteoglycans (NCAN, ACAN, BCAN) and neuroprotective proteins (CDH8, HSPB1, ADIPOQ), suggesting impaired neurodevelopment and reduced stress response capacity. Additionally, increased levels of injury-associated fatty acid-binding proteins (FABPs) were observed. These findings support the role of urinary proteomics as a noninvasive approach to identify early biomarkers of preterm brain injury and to uncover underlying pathophysiological mechanisms. Further investigation of the identified biomarkers in vitro or in animal models would be a valuable next step to better understand their functional relevance and potential clinical applicability.

## Clinical Perspectives

1. Background – Why the study was undertaken: Preterm infants, especially those born very early, are at high risk for brain injuries that can lead to lifelong neurological problems. Current tools often miss early signs of damage. This study explored whether urine - a simple, noninvasive sample - could help detect these injuries sooner.
2. Brief summary of the results: We found specific protein patterns in the urine of preterm infants with brain injury. Protective proteins were lower, while proteins linked to inflammation and brain damage were higher. These changes reflect underlying disruptions in brain development and inflammation.
3. Potential significance to human health and disease: Urinary proteomics could become a practical tool to identify preterm infants at risk of brain injury early on. This may enable earlier interventions, better monitoring, and more personalized care - potentially improving outcomes for vulnerable newborns.

## Supplementary Material



## Data Availability

The mass spectrometry proteomics data have been deposited to the ProteomeXchange Consortium via the PRIDE partner repository with the data set identifier PXD064065.

## Notes

Supporting Table S1 (.xlsx): Differentially regulated urinary proteins on day 1 of the observation period. Statistically significant proteins with altered urinary concentrations in premature infants with moderate/severe brain injury compared to those without brain injury. Supporting Table S2 (.xlsx): Differentially regulated urinary proteins on day 2 of the observation period. Statistically significant proteins with altered urinary concentrations in premature infants with moderate/severe brain injury compared to those without brain injury. Supporting Table S3 (.xlsx): Differentially regulated urinary proteins on day 3 of the observation period. Statistically significant proteins with altered urinary concentrations in premature infants with moderate/severe brain injury compared to those without brain injury. Supporting Table S4 (.xlsx): Differentially regulated urinary proteins on day 4 of the observation period. Statistically significant proteins with altered urinary concentrations in premature infants with moderate/severe brain injury compared to those without brain injury. Supporting Table S5 (.xlsx): Differentially regulated urinary proteins on day 6 of the observation period. Statistically significant proteins with altered urinary concentrations in premature infants with moderate/severe brain injury compared to those without brain injury. Supporting Table S6 (.xlsx): Differentially regulated urinary proteins on day 8 of the observation period. Statistically significant proteins with altered urinary concentrations in premature infants with moderate/severe brain injury compared to those without brain injury. Supporting Table S7 (.xlsx): Differentially regulated urinary proteins on day 28 of the observation period. Statistically significant proteins with altered urinary concentrations in premature infants with moderate/severe brain injury compared to those without brain injury. Supporting Table S8 (.xlsx): Differentially regulated urinary proteins at term-equivalent age (TEA). Statistically significant proteins with altered urinary concentrations in premature infants with moderate/severe brain injury compared to those without brain injury (XLSX)

## Notes

This study protocol was reviewed and approved by the Jagiellonian University Bioethical Committee, Krakow, Poland (approval number 1072.6120.336.2020). Written informed consent for participation in the study was obtained from the participants’ parents.
